# Social media practices, risk perceptions, and policy awareness among dental practitioners and trainees in Saudi Arabia: a cross-sectional study

**DOI:** 10.3389/fmed.2026.1884234

**Published:** 2026-06-24

**Authors:** Moayad Othman, Salah Alshagrawi

**Affiliations:** 1 Department of Dentistry, Dr. Sulaiman Al Habib Medical Group, Riyadh, Saudi Arabia; 2 Department of Public Health, Saudi Electronic University, Riyadh, Saudi Arabia

**Keywords:** cross-sectional study, dental practitioners, e-professionalism, policy awareness, risk perception, Saudi Arabia, social media

## Abstract

**Background:**

Social media has become an important component of healthcare practice, offering opportunities for communication, education, and professional engagement while introducing risks related to patient confidentiality, professional conduct, and institutional reputation. Evidence on social media practices and e-professionalism among dental practitioners in Saudi Arabia remains limited.

**Objective:**

This study aimed to assess social media practices, risk perceptions, and policy awareness among dental practitioners and trainees in Saudi Arabia and to identify factors associated with variations in awareness and behavior.

**Methods:**

A cross-sectional online survey was conducted among 326 dental practitioners and trainees across Saudi Arabia. The questionnaire assessed social media use, risk perception using five Likert-scale items, and awareness of institutional and international guidelines. Responses were measured using a 5-point Likert agreement scale. The risk perception scale demonstrated acceptable internal consistency (Cronbach’s α = 0.73). Data were analyzed using descriptive statistics, chi-square tests, and binary logistic regression.

**Results:**

Instagram was the most commonly used platform (70.6%), and one-third of participants reported daily professional use. Risk perception was generally low, with a mean composite score of 2.88 (SD = 0.44); only 2.5% of participants had high risk perception. Awareness of guidelines was limited, with 38.0% reporting awareness of WHO guidelines, 25.2% UNESCO guidelines, and 35.9% Ministry of Health policies. Only 20.6% reported having a workplace social media policy, and 4.6% had received formal training. Although age and years of professional experience were associated with policy awareness in bivariate analyses (*p* < 0.001), only years of professional experience remained independently associated with policy awareness after adjustment. Practitioners with ≥5 years of experience were significantly less likely to have low policy awareness (AOR = 0.41; 95% CI, 0.22–0.76; *p* = 0.004).

**Conclusion:**

Social media use is widespread among dental practitioners in Saudi Arabia; however, awareness of formal guidelines and institutional policies remains limited. Greater professional experience was associated with higher policy awareness, suggesting that early-career practitioners may benefit from targeted training. Strengthening education on e-professionalism and implementing clear institutional policies are essential to support the safe and ethical use of social media in dental practice.

## 1 Introduction

Social media has fundamentally altered the ways in which individuals communicate, exchange information, and engage with professional communities. While social media offers substantial opportunities for professional communication, education, and public engagement, it also introduces ethical, legal, and professional challenges that require careful management (1, 2). The concept of “e-professionalism”—the expression of traditional medical professionalism within digital environments—has emerged as a critical concern for educators and regulators (3). In practice, the boundaries are often blurred, as personal posts may be perceived as professional endorsements and informal online interactions may be interpreted as clinical advice (4, 5). Consequently, actions in digital spaces can have immediate implications for professional standing.

Globally, healthcare professionals increasingly use social media for learning, networking, and professional development (6, 7). However, these benefits are accompanied by risks and require careful and responsible use (8). This dynamic is particularly evident in Saudi Arabia, where social media penetration is among the highest globally and platforms are widely used for health-related communication. Studies have reported a high prevalence of social media use for medical consultation and health information among the general population (9), with increased reliance during the COVID-19 pandemic (10, 11). Healthcare professionals in the Kingdom similarly integrate social media into their professional activities, using it for knowledge sharing, professional development, and safety awareness (12–14).

Within this context, dentistry represents a particularly sensitive domain. The visual and service-oriented nature of dental practice makes platforms such as Instagram, Facebook, and X powerful tools for patient engagement and practice promotion (15). Dentists frequently share clinical cases, particularly aesthetic outcomes, to attract and inform patients (16). In Saudi Arabia, such visual content has been shown to influence public perceptions of treatment needs and decisions regarding cosmetic dental interventions (17, 18). While this visibility supports professional growth, it simultaneously increases the risk of ethical violations. Sharing clinical images may inadvertently compromise patient privacy, and interactive platforms may encourage unsolicited advice, potentially exposing practitioners to legal liability and blurring professional boundaries (19). These risks are further amplified by the absence of formal training and the rapid pace of digital adoption relative to the development of regulatory guidelines (20).

Although social media use among healthcare professionals has been examined in Saudi Arabia, dental practitioners are a particularly important subgroup, as dental practice often involves visual case presentations, promotion of aesthetic treatments, and online patient engagement (21). Despite these risks, empirical evidence on Saudi dental practitioners’ social media behaviors, perceived risks, and awareness of institutional or national social media policies remains limited (22).

Given the limited evidence available in Saudi Arabia, further research is needed to better understand social media practices, risk perceptions, and policy awareness among dental practitioners and trainees. Therefore, this study aimed to assess social media practices, risk perceptions, and policy awareness among dental practitioners and trainees in Saudi Arabia. Specifically, it sought to evaluate patterns of social media use, identify perceived professional and ethical risks, assess awareness of relevant policies and guidelines, and examine whether demographic and professional characteristics are associated with variations in these outcomes.

This study is also informed by the concept of transversal competencies in health professions education. Within this framework, competencies such as professionalism, ethical decision-making, communication, digital literacy, and professional accountability are considered transferable capabilities that support effective practice across diverse clinical and professional contexts (23). Because social media use increasingly forms part of contemporary healthcare communication and professional engagement, e-professionalism may be viewed as an important manifestation of these competencies in digital environments. Examining risk perception, policy awareness, and training experiences among dental practitioners and trainees may therefore provide insight into how these transversal competencies are developed and applied within professional practice.

Based on existing literature, three hypotheses were formulated: (H1) social media use is highly prevalent among dental practitioners and trainees in Saudi Arabia; (H2) awareness of professional guidelines and institutional policies related to social media use is limited; and (H3) licensed practitioners and individuals with greater professional experience demonstrate higher levels of risk perception and policy awareness than trainees and less experienced practitioners.

## 2 Materials and methods

### 2.1 Study setting and data collection

This research employed a descriptive, cross-sectional study design. This approach was selected as the most appropriate and efficient method for assessing the prevalence of social media practices, risk perceptions, and policy awareness at a single point in time, without manipulating any variables. Data collection was conducted via a self-administered online questionnaire to ensure broad participation among dental professionals nationwide. The recruitment and data collection period lasted 4 weeks, from February 15, 2025, to March 15, 2025. A non-probability convenience sampling approach was used, supplemented by snowball sampling to increase reach across different regions and professional groups. The survey was disseminated across predefined professional groups based on geographic regions: Central region (Riyadh); Western region (Makkah and Madinah); Eastern region (Dammam); and Southern region (Abha); and employment sector [public sector (Ministry of Health hospitals) and private sector (private clinics and hospitals)] to enhance diversity through professional networks and associations. Subsequently, a snowball component was incorporated, in which participants were encouraged to share the survey link with their professional colleagues, thereby further expanding the sample’s reach. As a non-probability sampling method was employed, the findings should be interpreted with caution, and generalizability to the broader population of dental practitioners in Saudi Arabia may be limited.

The survey was distributed through multiple recruitment channels, including the Saudi Dental Society, professional social media groups, and participant-driven snowball sharing. Because the survey link was disseminated simultaneously across these channels and could be further shared by participants, the specific recruitment source for individual respondents was not recorded. Consequently, the relative contribution of each recruitment channel could not be determined. Furthermore, the total number of individuals who received or viewed the survey invitation was unknown, and a conventional response rate could not be calculated.

Ethical approval for this study was obtained from the Research Ethics Committee (REC) at the Saudi Electronic University (CHS-202510-014). Informed consent was obtained from all participants via a mandatory initial step in the online questionnaire. Before accessing the survey questions, participants were presented with a detailed digital consent form that outlined the study’s purpose, procedures, risks, benefits, and confidentiality measures.

### 2.2 Participants

Study participants were selected based on predefined inclusion and exclusion criteria. Eligible participants included individuals currently enrolled as dental students (4th year and above), interns, or practicing dental professionals, including general dentists, specialists, consultants, and postgraduate residents. Participants were also required to be currently living in and/or working in Saudi Arabia, to possess at least one active social media account, and to provide informed electronic consent to participate in the study.

Individuals who were not dental students or qualified dental practitioners (e.g., dental assistants, hygienists, and technicians) were excluded, as were dental practitioners currently residing or practicing outside Saudi Arabia, individuals who did not use any form of social media, and those who did not complete the informed consent section of the questionnaire. Participants were recruited through professional networks, including the Saudi Dental Society, and through professional social media groups dedicated to dental professionals in Saudi Arabia. As this was a cross-sectional study, no follow-up was conducted.

### 2.3 Study population and sample size determination

The target population for this study is all eligible dental practitioners in Saudi Arabia. The sample size was calculated using the single-population proportion method for cross-sectional studies, a standard approach for estimating a minimum sample size when the outcome variable is categorical (24). A 95% confidence level (*Z* = 1.96), an estimated proportion (p) of 0.5 (to yield the maximum sample size and ensure adequacy), and a margin of error (e) of 0.05 were used. Based on these assumptions, the minimum required sample size was approximately 318 participants. Ultimately, 326 participants completed the survey, exceeding the predefined target and providing sufficient data for analysis.

### 2.4 Study variables

The primary outcome variables included social media practices, risk perception, and policy awareness. Social media practices were measured by frequency of use, platforms used, nature of content shared, and types of patient interactions online. Risk perception was assessed using Likert-scale questions about the perceived likelihood and severity of legal, ethical, and professional risks. Responses were measured using a 5-point Likert-scale agreement (1 = strongly disagree, 5 = strongly agree), with higher scores indicating greater agreement with each statement. A composite risk perception score was calculated as the mean of the five risk perception items (range: 1–5). For inferential analyses, participants with a composite score of 4 or higher were classified as having high-risk perception. Because no established cut-off values exist for this adapted measure, this threshold was selected to identify participants who generally agreed or strongly agreed with statements reflecting awareness of professional, ethical, and legal risks associated with social media use.

Policy awareness was measured by awareness of existing institutional policies, understanding of their content, and attitudes toward policy implementation. Policy awareness was assessed using three binary (Yes/No) questions evaluating participants’ awareness of the World Health Organization (WHO) guidance on digital professionalism, the United Nations Educational, Scientific and Cultural Organization (UNESCO) guidance on digital ethics, and the Saudi Ministry of Health (MOH) social media policy. A composite policy awareness score ranging from 0 to 3 was calculated by assigning one point for each guideline or policy recognized by the participant. Participants scoring 0–1 were classified as having low policy awareness, whereas those scoring 2–3 were classified as having high policy awareness. This threshold was selected to distinguish participants demonstrating awareness of at least two of the three assessed guidelines or policies from those with awareness of none or only one, thereby providing a practical indicator of broader policy awareness.

Predictor variables included demographic and professional characteristics, such as age, gender, years of experience, professional title (e.g., student, general dentist, specialist), sector of employment (public/private), and geographic region. Age and years of experience were considered as potential confounders in the relationship between professional status and risk perception.

### 2.5 Measuring tool/instrument

The data collection instrument was a structured online questionnaire developed and administered using the Google Forms platform. The questionnaire was designed through a comprehensive multi-step process. First, existing validated instruments examining social media use, e-professionalism, and online professional conduct among healthcare students and professionals were reviewed and adapted to ensure content validity and relevance to the dental profession (6, 25, 26). Second, the initial draft was reviewed by a panel of three experts in public health, dental ethics, and research methodology to assess face and content validity, and their feedback was used to refine the items’ clarity, relevance, and comprehensiveness. The questionnaire was administered exclusively in English, the primary language of instruction in Saudi dental schools and postgraduate dental training programs, and widely used in professional and academic dental communication. Therefore, English proficiency was considered sufficient among the target population of dental students, interns, residents, and practicing dental professionals. Responses were measured using a 5-point Likert-scale agreement (1 = strongly disagree, 5 = strongly agree). The final questionnaire consisted of four sections: digital informed consent, demographic and professional characteristics, social media practices (usage patterns and behaviors), and risk perceptions and policy awareness. To ensure reliability and validity, a pilot study was conducted with a convenience sample of 20 dental professionals who met the inclusion criteria but were excluded from the final analysis. The pilot assessed clarity and understandability, used feedback to revise ambiguous items, evaluated internal consistency using Cronbach’s alpha (α > 0.7 considered acceptable), and found that the average completion time was approximately 10–15 min.

To further examine the dimensionality of the risk perception scale, an exploratory factor analysis (EFA) was conducted using principal axis factoring. Sampling adequacy was assessed using the Kaiser-Meyer-Olkin (KMO) measure and Bartlett’s test of sphericity. The EFA was performed to evaluate the underlying factor structure and support the construct validity of the adapted risk perception scale. Factors with eigenvalues greater than 1.0 were retained, and factor loadings of 0.40 or higher were considered acceptable indicators of item-factor association.

### 2.6 Statistical methods

Based on these findings, variables were summarized using means and standard deviations or medians and interquartile ranges, as appropriate, and continuous variables were categorized only when required for specific analyses, such as Chi-square testing. All statistical analyses were performed using IBM SPSS Statistics (Version 29.0). Descriptive statistics were used to summarize all variables, including frequencies, percentages, means, and standard deviations. Prior to inferential analysis, the normality of continuous variables (e.g., age and years of experience) was assessed using both the Kolmogorov-Smirnov and Shapiro-Wilk tests. Based on these results, continuous variables were described and handled appropriately, with means and standard deviations used for normally distributed data and medians and interquartile ranges for non-normally distributed data. For analyses requiring categorization (e.g., Chi-square tests), continuous variables were grouped into meaningful categories (e.g., age groups: 21–25 years, 26–30 years, 31–35 years, and ≥ 36 years). Due to the small number of postgraduate residents (*n* = 12), they were combined with interns for inferential analyses to improve statistical stability and satisfy the expected cell count assumption. Fisher’s exact test was used when expected cell counts were fewer than 5. Bivariate analyses were conducted using Chi-square tests to examine associations between categorical predictor variables (e.g., professional title) and outcome variables (e.g., high vs. low-risk perception). Independent *t*-tests or ANOVA were used for continuous outcomes where appropriate.

Multivariable analysis was performed using binary logistic regression to identify independent predictors of the primary outcomes while controlling for potential confounders. Variables considered for inclusion in the model included age group, gender, professional status, years of professional experience, geographic region, and practice area. Variables showing an association with policy awareness in the bivariate analyses (*p* < 0.20), as well as variables considered theoretically relevant based on prior literature, were entered into the initial model. A backward stepwise likelihood ratio procedure was used to identify independent predictors of policy awareness. Multicollinearity was assessed using variance inflation factors (VIFs), with values below 5.0 considered indicative of acceptable multicollinearity. Particular attention was given to the age group and years of professional experience due to their conceptual overlap. Results were reported as adjusted odds ratios (AORs) with 95% confidence intervals (CIs). A *p*-value of less than 0.05 was considered statistically significant for all analyses.

To address the potential influence of geographic overrepresentation, additional regional subgroup analyses were conducted by comparing the main outcome variables across Makkah, Riyadh, and other regions. Chi-square tests or Fisher’s exact tests were used for categorical outcomes, and one-way ANOVA or Kruskal-Wallis tests were used for continuous outcomes, as appropriate. Region was also considered in the multivariable logistic regression model to assess whether geographic location independently influenced policy awareness.

## 3 Results

### 3.1 Participant characteristics

A total of 326 dental practitioners and trainees completed the survey (Table 1). The sample comprised 174 females (53.4%) and 152 males (46.6%), with a mean age of 30.5 years (SD = 9.9). The largest professional group was undergraduate students (42.9%), followed by specialists/consultants (29.8%) and general dentists (17.8%). Most respondents were from Makkah (64.4%) and Riyadh (25.8%). Among the 168 licensed practitioners included in the study, 111 (66.1%) reported five or more years of professional experience, while 57 (33.9%) reported fewer than 5 years of experience.

**TABLE 1:** Demographic and professional characteristics (*N* = 326).

Variable	*n*	%
Gender		
Female	174	53.4
Male	152	46.6
Age group		
21–25 years	147	45.1
26–30 years	57	17.5
31–35 years	41	12.6
≥ 36 years	81	24.8
Professional status		
Undergraduate student	140	42.9
Intern	19	5.8
Postgraduate resident	12	3.7
General dentist	58	17.8
Specialist/consultant	97	29.8
Region		
Makkah	210	64.4
Riyadh	84	25.8
Other regions	32	9.8
Years of experience		
< 5 years	57	33.9
≥ 5 years	111	66.1
Practice setting		
Governmental	73	43.5
Private	46	27.4
Both	49	29.2

### 3.2 Social media usage patterns

Instagram was the most widely used platform (70.6%), followed by X (62.0%) and Snapchat (53.7%). One third of respondents used social media daily for professional purposes (33.7%), and another third used it rarely (33.7%). Positive attitudes toward social media as a professional tool were prevalent: mean scores for “improves professional communication” (3.91/5) and “helps improve clinical skills” (3.90/5) were high. The majority (61.3%) reported always obtaining patient consent before sharing identifying photographs, while 19.0% reported sometimes, rarely, or never doing so. A statistically significant gender difference was observed in consent-seeking behavior (χ^2^ = 14.109, df = 4, *p* = 0.007), with females more likely to always seek consent. Online behavior patterns showed that publishing self-identifying professional photographs was considered professional by 75.8%; 28.5% had personally done so. 47.5% viewed publishing photographs of colleagues in clinical settings as unprofessional; 22.1% admitted to having done it, and 77.6% had seen such content online.

### 3.3 Risk perception

Risk perception was generally low across all five items, as reflected by a mean composite risk perception score of 2.88 (SD = 0.44); only 8 participants (2.5%) met the predefined threshold for high-risk perception (composite score ≥4) (Table 2). This finding suggests limited awareness among dental practitioners and trainees of the potential legal, ethical, and professional risks associated with social media use and is explored further in the Discussion section. The internal consistency of the five-item scale was acceptable (Cronbach’s α = 0.73). Exploratory factor analysis further supported the use of the composite risk perception score. The Kaiser–Meyer–Olkin measure demonstrated acceptable sampling adequacy (KMO = 0.71), and Bartlett’s test of sphericity was statistically significant (χ^2^ = 214.6, *p* < 0.001), indicating that the data were suitable for factor analysis. A single factor with an eigenvalue greater than 1.0 was extracted, explaining 48.3% of the total variance. Factor loadings ranged from 0.51 to 0.79, supporting a unidimensional structure of the scale.

**TABLE 2:** Descriptive statistics for risk perception items (*N* = 326).

Item	Mean (SD)	Agree/strongly agree *n* (%)	Disagree/strongly disagree *n* (%)
Personal posts can harm public trust in the workplace	2.82 (0.90)	60 (18.4)	144 (44.2)
A healthcare facility could be held legally responsible	2.86 (0.91)	67 (20.6)	136 (41.7)
A patient discovering a practitioner’s profile could harm the therapeutic relationship.	2.82 (0.90)	62 (19.0)	142 (43.6)
Fair for employers to review SM before hiring	2.84 (0.92)	62 (19.0)	141 (43.3)
Confident I understand the career risks of SM use	3.06 (1.05)	99 (30.4)	121 (37.1)
Composite risk score	2.88 (0.44)	–	–

The composite risk perception score is presented as a mean (SD) because it represents the average of five Likert-scale items; therefore, no frequency or percentage is reported.

### 3.4 Policy awareness

Awareness of guidelines was low and marked by uncertainty (Table 3). Only 38.0% knew the WHO guidelines, 25.2% the UNESCO guidelines, and 35.9% the MOH e-health policies. At the workplace level, only 20.6% reported a formal social media policy, and 4.6% of the total sample had received training on it. The composite policy awareness score (range 0–3) had a mean of 0.99; 30.4% scored ≥ 2 (high awareness).

**TABLE 3:** Awareness of social media guidelines (*N* = 326).

Guideline source	Yes *n* (%)	No *n* (%)	Not sure *n* (%)
WHO	124 (38.0)	62 (19.0)	140 (43.0)
UNESCO	82 (25.2)	80 (24.5)	164 (50.3)
MOH	117 (35.9)	55 (16.9)	154 (47.2)

### 3.5 Predictors of risk perception and policy awareness

Chi-square tests (Table 4) showed that years of experience and age group were significantly associated with high-risk perception (*p* < 0.01). Significant differences in high-risk perception across professional status categories remained evident after category consolidation. The intern/postgraduate resident group demonstrated the highest proportion of high-risk perception (12.9%), compared with undergraduate students (0.7%), general dentists (1.7%), and specialists/consultants (2.1%). Gender, region, and practice area were not significant. Due to small subgroup sizes and violations of Chi-square assumptions (expected cell counts ≥5) for some comparisons, these results should be interpreted with caution. Cramér’s V effect sizes are interpreted as: small ( ≥ 0.10), medium ( ≥ 0.30), large ( ≥ 0.50). For inferential analyses, the intern and postgraduate resident categories were combined into a single group to meet the minimum expected cell count assumptions for chi-square testing.

**TABLE 4:** Association between professional status and high-risk perception.

Professional status	High risk perception *n* (%)	Low risk perception *n* (%)	Total *n*	*p*-value
Undergraduate student	1 (0.7)	139 (99.3)	140	χ^2^ = 51.57, *p* < 0.001
Intern/postgraduate resident*	4 (12.9)	27 (87.1)	31	
General dentist	1 (1.7)	57 (98.3)	58	
Specialist/consultant	2 (2.1)	95 (97.9)	97	
Total	8 (2.5)	318 (97.5)	326	

*Combined into a single category for inferential analyses because of small subgroup sizes.

For policy awareness, all predictors except gender were significant (Table 5). Years of experience had the strongest effect (χ^2^ = 70.931, *p* < 0.001, *V* = 0.466). Specialists/consultants showed the highest awareness (56.7%), while undergraduate students (9.3%) and postgraduate residents (0%) had very low awareness. Age and years of experience were also strong predictors. Region and practice areas were also significant (*p* < 0.05).

**TABLE 5:** Association between professional status and high policy awareness.

Professional status	High policy awareness *n* (%)	Low policy awareness *n* (%)	Total *n*	*p*-value
Undergraduate student	13 (9.3)	127 (90.7)	140	χ^2^ = 70.93, *p* < 0.001
Intern/postgraduate resident	2 (6.5)	29 (93.5)	31	
General dentist	29 (50.0)	29 (50.0)	58	
Specialist/consultant	55 (56.7)	42 (43.3)	97	
Total	99 (30.4)	227 (69.6)	326	

The intern and postgraduate resident categories were combined due to small subgroup sizes. Fisher’s exact test was considered when expected cell counts were below five.

The initial multivariable model included age group, gender, professional status, years of professional experience, geographic region, and practice area. After adjustment, years of professional experience remained the only statistically significant predictor of policy awareness. Age group, gender, professional status, geographic region, and practice area were not independently associated with policy awareness and did not remain significant in the final model.

The results of the multivariable logistic regression analysis are presented in Table 6. After adjustment for potential confounders, years of experience remained the only independent predictor of policy awareness. Because the regression model used low policy awareness as the outcome variable, an adjusted odds ratio below 1.0 indicates a reduced likelihood of low awareness. Practitioners with ≥5 years of experience were significantly less likely to demonstrate low policy awareness (AOR = 0.41, 95% CI: 0.22–0.76, *p* = 0.004), indicating higher levels of policy awareness than those with fewer than 5 years of experience. No significant associations were observed for gender, age group, professional status, or geographic region after adjustment. The Hosmer–Lemeshow test indicated good model fit (χ^2^ = 8.14, df = 8, *p* = 0.42), and the model explained 12% of the variance in policy awareness (Nagelkerke *R*^2^ = 0.12).

**TABLE 6:** Binary logistic regression analysis of factors associated with low policy awareness.

Variable	Crude OR (95% CI)	*p*-value	Adjusted OR (95% CI)	*p*-value
Female (ref: Male)	1.08 (0.67–1.73)	0.75	1.01 (0.61–1.68)	0.97
Age ≥ 36 years (ref: 21–25 years)	0.42 (0.24–0.72)	0.002	0.71 (0.35–1.43)	0.34
Specialist/consultant (ref: undergraduate student)	12.84 (6.41–25.73)	< 0.001	1.88 (0.71–4.96)	0.20
General dentist (ref: undergraduate student)	9.74 (4.53–20.92)	< 0.001	1.41 (0.56–3.55)	0.47
≥ 5 years experience (ref: < 5 years)	0.21 (0.12–0.38)	< 0.001	0.41 (0.22–0.76)	0.004
Riyadh (ref: Makkah)	1.22 (0.73–2.04)	0.45	1.10 (0.62–1.94)	0.74
Other regions (ref: Makkah)	1.08 (0.50–2.32)	0.85	0.98 (0.43–2.24)	0.96

Model statistics: Hosmer–Lemeshow χ^2^ = 8.14, df = 8, *p* = 0.42, Nagelkerke *R*^2^ = 0.12; CI = Confidence Interval. The dependent variable was low policy awareness (coded as 1 = low awareness and 0 = high awareness). Therefore, adjusted odds ratios (AORs) below 1.0 indicate lower odds of low policy awareness and correspondingly higher levels of policy awareness.

Assessment of multicollinearity showed no evidence of problematic correlation among the predictor variables. The VIF values for age groups and years of professional experience were 2.31 and 2.48, respectively, both well below the predefined threshold of 5.0. These findings indicate that multicollinearity was not a significant concern and suggest that the loss of statistical significance for age in the adjusted model was unlikely to be attributable to excessive collinearity.

### 3.6 Regional subgroup analysis

To assess whether the geographical overrepresentation of participants from Makkah influenced the study findings, additional subgroup analyses were conducted comparing participants from Makkah, Riyadh, and other regions. The analyses examined differences in social media practices, risk perception, and policy awareness across regions. No statistically significant regional differences were observed in the primary outcome variables, including composite risk perception scores, high-risk perception, social media usage patterns, awareness of social media guidelines, or overall policy awareness (all *p* > 0.05). Furthermore, region was not significantly associated with high-risk perception or policy awareness in bivariate analyses. These results highlight that, despite the uneven geographic distribution of the sample, the main outcomes were relatively consistent across regions and were unlikely to be substantially influenced by regional institutional context.

## 4 Discussion

This study provides empirical evidence on social media e-professionalism among dental practitioners and trainees in Saudi Arabia. The findings confirm that social media is deeply embedded in professional life, yet risk perception is critically low and policy awareness is fragmented.

Although both age and years of professional experience were associated with policy awareness in the bivariate analyses, only years of professional experience remained a significant independent predictor in the multivariable model. This finding suggests that the apparent effect of age may be explained by its relationship with professional experience rather than representing an independent association with policy awareness.

An additional finding of interest was the absence of significant gender differences in either risk perception or policy awareness. Although some previous studies have reported gender-based differences in online behavior, privacy concerns, and professional social media use, our findings suggest that awareness of social media-related professional risks and policies may be relatively similar among male and female dental practitioners and trainees in Saudi Arabia. One possible explanation is that professional training environments, regulatory expectations, and patterns of social media use are increasingly shared across genders within contemporary dental education and practice. Nevertheless, further research is warranted to determine whether this finding is specific to the current sample or reflects broader trends within the dental profession.

The pervasive use of visual platforms (e.g., Instagram, Snapchat) aligns with dentistry’s nature as a visual specialty. The high rates of daily professional use (33.7%) and positive attitudes toward social media as a communication and educational tool are consistent with international studies (26). However, the substantial minority who rarely or never use social media professionally (33.7%) suggests a bimodal distribution that may reflect generational or practice-type differences. The most concerning finding is the extremely low risk perception: only 2.5% of participants demonstrated high risk awareness. This is despite documented risky behaviors, including sharing clinical photographs without clear de-identification (22.1% admitted to publishing colleagues’ clinical photos, 77.6% had seen such content). Policy awareness was similarly insufficient. Fewer than 40% were aware of any guideline, and workplace policies were nearly absent. The fact that policy awareness increases with years of experience suggests that awareness may be acquired informally through clinical exposure rather than through systematic training. This is problematic because early-career practitioners—who are often the most active social media users—are left without guidance. Although differences in risk perception were observed across professional groups, some subgroup sizes were small, particularly among postgraduate residents. Therefore, these findings should be interpreted cautiously and considered exploratory. Additional studies with larger, more balanced samples are needed to determine whether these observed differences reflect true variations in risk perception across professional groups.

The psychometric evaluation conducted in this study provided additional support for the use of the composite risk perception measure. Exploratory factor analysis indicated a unidimensional structure, and the scale demonstrated acceptable internal consistency. Nevertheless, further validation of the instrument in larger and more diverse dental populations is warranted. Additional regional subgroup analyses were conducted to assess whether the overrepresentation of participants from Makkah influenced the study findings. No statistically significant regional differences were observed in social media practices, risk perception, or policy awareness. Furthermore, region was not significantly associated with the primary outcome variables in the bivariate analyses. These findings suggest that, despite the uneven geographic distribution of the sample, the observed patterns were relatively consistent across regions and are unlikely to be substantially explained by regional institutional context.

From a health professions education perspective, the findings of this study can be interpreted through the lens of transversal competencies, which are increasingly recognized as essential outcomes of competency-based education (27). Transversal competencies refer to transferable capabilities that support effective professional practice across diverse contexts and include professionalism, ethical decision-making, communication, digital literacy, critical thinking, reflective practice, and professional accountability (28). Within contemporary healthcare environments, where digital communication and social media engagement are increasingly integrated into professional activities, e-professionalism can be viewed as the practical application of these competencies in online settings.

The low levels of risk perception and policy awareness observed in this study may be consistent with challenges in the acquisition and development of these competencies during dental education and early professional training, although these educational processes were not directly assessed in the present study (29). Although participants reported frequent social media use and generally positive attitudes toward its professional value, awareness of the ethical, legal, and professional implications of online behavior remained limited. This discrepancy may indicate that frequent engagement with digital platforms does not necessarily correspond to greater awareness of their professional, ethical, and regulatory implications, a phenomenon that has been discussed in the digital professionalism literature (28). In particular, limited awareness of professional guidance and institutional policies may reflect broader challenges related to ethical reasoning, professional accountability, and digital professionalism, although these constructs were not directly measured in the present study (30).

The finding that policy awareness increased with years of professional experience provides additional insight into how these competencies may develop. One possible explanation is that aspects of digital professionalism may develop through workplace exposure, mentorship, and accumulated professional experience, as suggested in previous health professions education literature, although these learning pathways were not directly examined in the present study (31). While experiential learning is valuable, reliance on informal learning pathways may lead to inconsistent competency development among practitioners and across training environments (23).

These findings have important implications for competency-based dental education. E-professionalism should not be viewed solely as a matter of policy compliance or risk management but as an educational outcome that can be taught, practiced, assessed, and reinforced throughout professional training. Educational strategies such as case-based learning, reflective exercises, structured discussions of ethical dilemmas, digital professionalism workshops, and competency-based assessments may help strengthen the transversal competencies required for safe, ethical, and effective participation in contemporary healthcare systems (20). Greater attention to these competencies may also help ensure that future dental professionals are equipped to navigate the increasingly complex digital dimensions of patient care, professional communication, and public engagement.

Our findings are consistent with Cain et al. (25), who reported concerns regarding accountability and online professional conduct among pharmacy students. Similarly, Ellaway et al. (29) highlighted concerns regarding the online posting of unprofessional content by healthcare trainees, emphasizing the potential consequences of inappropriate social media use for professional reputation and public trust. In the Saudi context, our results are consistent with earlier studies on medical students’ attitudes and physicians’ professional social media use (32) and extend this evidence to the dental profession. The low policy awareness aligns with global patterns (1). However, it is particularly striking given Saudi Arabia’s Vision 2030 emphasis on digital transformation and quality (33). The findings suggest that integrating e-professionalism into dental curricula may help improve awareness of ethical and professional responsibilities associated with social media use. Structured case-based training on consent, de-identification, and professional boundaries may represent a useful strategy for improving awareness of professional responsibilities associated with social media use. Healthcare organizations may benefit from implementing clear social media policies and guidance documents to support professional online conduct and reduce uncertainty regarding acceptable digital practices. A substantial gap in policy awareness was observed among dental practitioners and trainees participating in this study. Awareness of international and institutional guidelines was limited, workplace social media policies were uncommon, and formal training on e-professionalism was rarely reported. As a result, many practitioners may rely primarily on personal judgment when engaging online, potentially increasing the risk of inconsistent professional practices. These findings highlight the need for greater integration of e-professionalism education within dental training programs, clearer institutional policies, and improved dissemination of existing professional guidance to support safe, ethical, and professional social media use. The present findings reinforce the importance of integrating transversal competencies into both dental education and continuing professional development. The observed deficiencies in risk perception, policy awareness, and formal training suggest that competencies related to professionalism, ethical decision-making, communication, digital literacy, and professional accountability may not be consistently developed or assessed across training pathways. Strengthening e-professionalism education may therefore contribute not only to safer social media use but also to broader competency development that supports high-quality, patient-centered, and ethically responsible dental practice in increasingly digital healthcare environments (34).

## 5 Conclusion

Social media use is widespread among dental practitioners and trainees in Saudi Arabia and is increasingly integrated into professional communication, education, and patient engagement. However, awareness of formal guidelines, institutional policies, and social media-related risks remains limited. While age and years of professional experience were associated with policy awareness in bivariate analyses, only years of professional experience remained an independent predictor after adjustment, suggesting that early-career practitioners may require additional support and guidance regarding responsible social media use.

These findings highlight the importance of strengthening education on e-professionalism, improving dissemination of professional guidance, and promoting awareness of institutional and international social media policies. Structured educational initiatives and clear organizational guidance may help support safer and more ethical online engagement among dental professionals.

The observed gaps in risk perception and policy awareness highlight the importance of strengthening transversal competencies related to digital professionalism, ethical decision-making, communication, and professional accountability within dental education and continuing professional development programs.

The gaps identified in policy awareness and risk perception extend beyond social media use and reflect broader challenges in professionalism, ethical decision-making, communication, and digital literacy in contemporary dental practice. Consequently, e-professionalism should be viewed as an important component of the transversal competencies required for modern dental practice. Strengthening these competencies is consistent with ongoing healthcare transformation efforts in Saudi Arabia and supports the development of responsible, ethical, and patient-centered healthcare professionals in an increasingly digital environment (35).

## 6 Limitations

Several limitations should be acknowledged. First, the study relied on self-reported data, which may be subject to recall bias and social desirability bias. Because the questionnaire included items related to potentially sensitive or professionally inappropriate behaviors, such as sharing clinical content without explicit consent or engaging in questionable social media practices, some participants may have underreported these behaviors to present themselves in a more favorable manner. Consequently, the prevalence of potentially risky social media practices reported in this study should be interpreted with caution and may represent conservative estimates of their true prevalence within the wider dental community.

Second, the use of convenience and snowball sampling may have introduced selection bias and limited the generalizability of the findings to all dental practitioners in Saudi Arabia. Because the survey was distributed through multiple online channels and participant-driven snowball sampling, the specific recruitment source for individual respondents was not recorded. Consequently, the relative contribution of each recruitment channel could not be assessed, and the total number of individuals who received the survey invitation was unknown. As a result, a conventional response rate could not be calculated, limiting assessment of potential recruitment-related selection bias and non-response bias.

Third, the geographic distribution of participants was uneven, with overrepresentation from Makkah and underrepresentation of other regions. In addition, the sample was disproportionately composed of younger practitioners and trainees. Because perceptions of professional and ethical risks may vary across career stages, this demographic distribution may have influenced the overall estimates of risk perception and policy awareness and may limit the generalizability of the findings to more experienced dental professionals. Although additional subgroup analyses did not identify significant regional differences in the primary outcome variables, the limited representation of some regions may have reduced the ability to detect smaller regional variations.

Another important limitation relates to the measurement instrument. Although the questionnaire was adapted from previously published instruments, reviewed by subject-matter experts, pilot-tested, and demonstrated acceptable internal consistency, it was not independently validated in a Saudi dental population. Furthermore, the questionnaire was administered exclusively in English, and no validated Arabic version was available. While English is widely used in dental education and professional practice in Saudi Arabia, the absence of a formally validated bilingual instrument may have influenced not only participation but also respondents’ interpretation of questionnaire items and Likert-scale response options, particularly among non-native English speakers. Consequently, the findings should be interpreted with appropriate caution, and future studies should consider developing and psychometrically validating both English and Arabic versions of the instrument for use in Saudi dental populations.

Although exploratory factor analysis supported the dimensionality of the risk perception scale, a comprehensive psychometric validation process was not performed. Confirmatory factor analysis, convergent validity, discriminant validity, and corrected item-total correlation analyses were beyond the scope of the present study. Consequently, the risk perception scale should be considered an adapted measure requiring further validation in future research.

Furthermore, the cross-sectional design precludes causal inference. The low prevalence of high-risk perception reduced statistical power for some subgroup analyses, and some professional subgroups contained relatively small numbers of participants, particularly postgraduate residents. Consequently, subgroup-specific estimates may be less stable and should be interpreted with caution. Although the achieved sample size exceeded the minimum requirement for estimating population proportions, the study was not specifically powered for multivariable logistic regression analyses. Therefore, the regression findings should be interpreted as exploratory and hypothesis-generating rather than confirmatory. Future studies employing probability-based sampling methods, balanced regional recruitment, and longitudinal designs are warranted to enhance representativeness and strengthen causal inference.

## Data Availability

The raw data supporting the conclusions of this article will be made available by the authors, without undue reservation.

## References

[1] Koo K Ficko Z Gormley E. Unprofessional online behavior among US medical students: a systematic review. *J Med Internet Res.* (2021) 23:e27056.

[2] Farnan J Snyder Sulmasy L Worster B Chaudhry H Rhyne J Arora V. Online medical professionalism: patient and public relationships: policy statement from the American college of physicians and the federation of state medical boards. *Ann Intern Med.* (2013) 158:620–7. 10.7326/0003-4819-158-8-201304160-00100 23579867

[3] Cain J Romanelli F. E- professionalism: a new paradigm for a digital age. *Curr Pharm Teach Learn.* (2019) 11:856–61.

[4] Gholami-Kordkheili F Wild V Strech D. The impact of social media on medical professionalism: a systematic qualitative review. *BMC Med Ethics.* (2022) 23:35. 23985172 10.2196/jmir.2708 PMC3758042

[5] Greysen S Kind T Chretien K. Online professionalism and the mirror of social media. *J Gen Intern Med.* (2010) 25:1227–9. 10.1007/s11606-010-1447-1 20632121 PMC2947638

[6] Alsobayel H. Use of social media for professional development by health care professionals: a cross-sectional web-based survey. *JMIR Med Educ.* (2016) 2:e15. 10.2196/mededu.6232 27731855 PMC5053809

[7] Grajales F Sheps S Ho K Novak-Lauscher H Eysenbach G. Social media: a review and tutorial of applications in medicine and health care. *J Med Internet Res.* (2014) 16:e13. 10.2196/jmir.2912 24518354 PMC3936280

[8] Alshakhs F Alanzi T. The evolving role of social media in health-care delivery: measuring the perception of health-care professionals in Eastern Saudi Arabia. *J Multidiscip Healthc.* (2018) 11:473–9. 30275699 10.2147/JMDH.S171538 PMC6157575

[9] Alyahya K Alhomaidhi R Al-Dhwaihy L Bin Semaih N Al-Bulushi N Al-Qahtani S et al. The prevalence of social media uses for medical consultation and health information in Saudi Arabia. *Int J Adv Appl Sci.* (2020) 7:63–7.

[10] Alotaibi K Alotiby A. Most common social media platforms in Saudi Arabia for sharing COVID-19 medical information. *Saudi J Health Syst Res.* (2022) 2:78–84.

[11] Alrasheedi R Alghnam S Almulhim A Alrasheedi F Alqahtani A. The role of social media in the public health response during the COVID-19 pandemic in Saudi Arabia: a cross-sectional study. *Front Public Health.* (2023) 11:1187423.

[12] Alanzi T Al-Habib D. The use of social media by healthcare quality personnel in Saudi Arabia. *J Environ Public Health.* (2020) 2020:1417478. 10.1155/2020/1417478 32565836 PMC7262735

[13] Attar R Almohanna A Almusharraf A Alhazmi A Alanzi N Al-Anezi F et al. Use of social media for the improvement of safety knowledge and awareness among Saudi Arabian phlebotomists. *Front Med.* (2023) 10:1194969. 10.3389/fmed.2023.1194969 37654654 PMC10466136

[14] Ventola C. Social media and health care professionals: benefits, risks, and best practices. *PT.* (2014) 39:491–520. PMC4103576 25083128

[15] Al Jawahry M. Digital engagement and patient acquisition in dental clinics: insights from UAE dentists. *Int J Appl Technol Med Sci.* (2025) 4:22–30. 10.54878/kytgbt66

[16] Aldossary MS Alqahtani AS Alasmari DS. Social media use and professional conduct among dental practitioners in Saudi Arabia: opportunities and ethical challenges. *BMC Oral Health.* (2023) 23:223–6.37072843

[17] Alalola B Alghanim L Almoneef S Aba Numay S Aldghim A Abu Wathlan J. The impact of orthodontists’ image-based social media posts on the public’s willingness to seek treatment in the central region of Saudi Arabia. *Patient Prefer Adherence.* (2025) 19:1255–61. 10.2147/PPA.S521608 40337287 PMC12057624

[18] Al-Khalifa K Al-Swuailem A AlSheikh R Muazen Y. Influence of social media on esthetic dental treatment decisions among Saudi adults. *Clin Cosmet Investig Dent.* (2022) 14:1–10.

[19] Pai S. Guardians of trust: the legal and ethical dimensions of protecting doctor-patient confidentiality in clinical practice. *Indian J Orthop.* (2025) 60:1172–6. 10.1007/s43465-025-01449-8 42221178 PMC13219538

[20] Chretien K Kind T. Social media and clinical care: ethical, professional, and social implications. *Circulation.* (2013) 127:1413–21. 10.1161/CIRCULATIONAHA.112.128017 23547180

[21] Wang Z Wang S Zhang Y Jiang X. Social media usage and online professionalism among registered nurses: a cross-sectional survey. *Int J Nurs Stud.* (2019) 98:19–26. 10.1016/j.ijnurstu.2019.06.001 31255853

[22] Kamarudin Y Mohd Nor N Libamin A Suriani A Marhazlinda J Bramantoro T et al. Social media use, professional behaviors online, and perceptions toward e-professionalism among dental students. *J Dent Educ.* (2022) 86:958–67. 10.1002/jdd.12912 35247218

[23] Frank J Snell L Cate O Holmboe E Carraccio C Swing S et al. Competency-based medical education: theory to practice. *Med Teach.* (2010) 32:638–45. 10.3109/0142159X.2010.501190 20662574

[24] Charan J Biswas T. How to calculate sample size for different study designs in medical research? *Indian J Psychol Med.* (2013) 35:121–6. 10.4103/0253-7176.116232 24049221 PMC3775042

[25] Cain J Scott D Akers P. Pharmacy students’ Facebook activity and opinions regarding accountability and e-professionalism. *Am J Pharm Educ.* (2009) 73:104. 10.5688/aj7306104 19885073 PMC2769526

[26] Henry R Molnar A Henry JC. A survey of US dental practices’ use of social media. *J Contemp Dent Pract.* (2012) 13:137–41. 10.5005/jp-journals-10024-1109 22665737

[27] Kind T Patel P Lie D Chretien K. Twelve tips for using social media as a medical educator. *Med Teach.* (2014) 36:284–90. 10.3109/0142159X.2013.852167 24261897

[28] Guckian J Hanson J Mak V. The future of professionalism: social media, digital professionalism and health professions education. *Med Educ.* (2021) 55:133–41.

[29] Ellaway R Coral J Topps D Topps M. Exploring digital professionalism. *Med Teach.* (2015) 37:844–9. 10.3109/0142159X.2015.1044956 26030375

[30] George D Dellasega C. Social media in medical education: two innovative pilot studies. *Med Educ.* (2011) 45:1158–9. 10.1111/j.1365-2923.2011.04124.x 21939449

[31] Cruess R Cruess S Steinert Y. Amending miller’s pyramid to include professional identity formation. *Acad Med.* (2016) 91:180–5. 10.1097/ACM.0000000000000913 26332429

[32] Alanzi T Al-Yami S. Physicians’ attitude towards the use of social media for professional purposes in Saudi Arabia. *Int J Telemed Appl.* (2019) 2019:6323962. 10.1155/2019/6323962 31885550 PMC6915001

[33] Health Sector Transformation Program. *Saudi Vision 2030.* (2026). Available online at: https://www.vision2030.gov.sa/en/explore/programs/health-sector-transformation-program (accessed December 22, 2025).

[34] World Health Organization. *Transforming and Scaling Up Health Professionals’ Education and Training: WHO Education Guidelines 2013.* Geneva: WHO (2013). 26042324

[35] Al-Luhaym A Alshagrawi S. Factors associated with access to the Saudi primary healthcare in light of Vision 2030. *Sudan J Med Sci.* (2023) 18:391–401. 10.18502/sjms.v18i3.14092

